# Evolving relationships between air pollution attenuation and AECOPD hospitalizations during a stringent control period in Shijiazhuang, China, 2017–2024

**DOI:** 10.3389/fpubh.2026.1741666

**Published:** 2026-02-10

**Authors:** Jingsi Cao, Zihan Ren, Xinyue Liu, Yanan Guo, Yunqian Wei, Xixin Yan

**Affiliations:** 1 The First Department of Pulmonary and Critical Care Medicine, The Second Hospital of Hebei Medical University, Hebei Key Laboratory of Respiratory Critical Care Medicine, Hebei Institute of Respiratory Diseases, Shijiazhuang, Hebei, China; 2 Department of Thoracic Surgery, Organ Transplantation Center, The First Hospital of Jilin University, Changchun, Jilin, China

**Keywords:** AECOPD hospitalizations, air pollution control, ecological study, respiratory health effects, time-series analysis

## Abstract

**Background:**

Shijiazhuang, a typical industrial city, faced severe air pollution that elevated AECOPD risk. The period 2017–2024 coincided with stringent air pollution control, providing a key window to examine the evolving relationship between air quality improvements and AECOPD hospitalizations.

**Methods:**

We analyzed 57,782 AECOPD hospitalizations (2017–2024) and air pollution data using a generalized additive Poisson time-series model. The analysis incorporated distributed lag non-linear terms while adjusting for meteorological conditions, seasonal trends, and day-of-week effects, with sensitivity analyses confirming result reliability.

**Results:**

PM_2.5_, PM10, and SO_2_ concentrations fell by 46%, 68%, and 85%, respectively. PM_2.5_-attributable excess cases under WHO standards dropped by 37.1%. Crucially, the association strength (relative risk) between most pollutants and hospitalizations showed a significant attenuating trend over time.

**Conclusion:**

During this stringent air pollution control period in Shijiazhuang, marked air quality improvements were associated with reduced AECOPD hospitalizations and attenuated pollutant-health associations. Persistent ozone pollution highlights the need for coordinated VOC-NO^x^ management.

## 1 Background

As a leading global consumer of fossil fuels, China's energy mix remains heavily dependent on coal. Coal combustion, particularly from the widespread use of decentralized coal heating in northern China during winter, is a major source of atmospheric pollution. This process releases substantial amounts of air pollutants, including fine particulate matter (PM_2.5_) and sulfur dioxide (SO_2_). These emissions drive a seasonal surge in air pollution during winter months, providing clear evidence of the significant link between coal combustion and atmospheric pollution (1). As the capital of Hebei Province and a core city in the Beijing-Tianjin-Hebei region, Shijiazhuang is geographically situated at 114°6′E, 38°3′N, in the transitional zone between the Taihang and Yanshan Mountains. This topography obstructs prevailing winds during autumn and winter, resulting in poor atmospheric dispersion conditions that exacerbate local air pollution.

Chronic Obstructive Pulmonary Disease (COPD) is a heterogeneous lung condition characterized by persistent airflow limitation and chronic respiratory symptoms (e.g., dyspnea, cough, sputum production), with its pathological basis involving abnormalities in the airways, alveoli, and pulmonary vasculature (2). The World Health Organization (WHO) predicts that COPD will become the third leading cause of death globally by 2030, affecting over 400 million people and posing a major public health challenge (3). Acute Exacerbation of COPD (AECOPD) constitutes the largest portion of the overall COPD burden (4). Previous studies have demonstrated that atmospheric pollutants significantly contribute to the hospitalization burden attributable to AECOPD in Shijiazhuang (5, 6).

In 2013, China launched the Air Pollution Prevention and Control Action Plan (APPCAP), implementing a series of stringent nationwide measures for comprehensive air quality management. By the end of 2017, the Beijing-Tianjin-Hebei region, as a key control area, had enforced the most rigorous pollution control measures (7). Notably, to address winter coal combustion pollution, the Chinese government introduced the Northern China Winter Clean Heating Plan (NCWCHP) in 2017, focusing on clean energy substitution in heavily polluted cities, including those in the Beijing-Tianjin-Hebei region and surrounding areas. Concurrent with the nationwide air quality improvements during this policy period, a decline in AECOPD hospitalizations has been observed in multiple studies. Research in Guangdong province linked air quality improvements to reduced AECOPD admissions (51), while a study in Jinan documented significant environmental, health, and economic benefits—including fewer AECOPD hospitalizations—from pollution control measures implemented between 2013 and 2017 (8). A study on the Beijing-Tianjin-Hebei region projected approximately 40% reductions in both PM_2.5_ and SO_2_ from 2012 to 2020 (9). A comparative analysis in Beijing showed that excess AECOPD cases attributable to PM_2.5_ exposure fell from 12,679 cases in 2013 to 7,377 in 2017, representing a substantial reduction in the disease burden driven by air quality improvements (10). Collectively, this evidence demonstrates that China's stringent air pollution policies have yielded substantial co-benefits, significantly reducing both AECOPD hospitalizations and mortality.

The APPCAP and the NCWCHP together established a sustained period of intensive multi-pollutant control. For cities like Shijiazhuang, identified as a “national hotspot for pollution exposure” (11), the 2017–2024 period represents a critical natural experiment window to investigate the health impacts of this concerted effort. While attributing health outcomes to specific policy instruments within such an integrated strategy is challenging, evaluating the overall health co-benefits coinciding with marked air quality improvements is of paramount public health importance.

To address this gap, this study establishes an 8-year (2017–2024) panel dataset for Shijiazhuang to systematically examine the evolving relationships between attenuation of multiple air pollutants and AECOPD hospitalizations during this stringent control period, thereby providing scientific evidence for the health co-benefits of integrated air quality management in similar industrial cities.

## 2 Methods

### 2.1 Data collection

AECOPD hospitalization data from January 1, 2017, to December 31, 2024, were obtained from the Shijiazhuang Medical Insurance Center. The data cover tertiary, secondary, and community hospitals within Shijiazhuang and include information such as age, gender, and admission date for all patients with a primary or secondary discharge diagnosis of AECOPD. Confidential patient identifiers (e.g., name, ID number, medical card number) were removed. The study's inclusion criteria were: (1) a discharge diagnosis of AECOPD based on the International Classification of Diseases, Ninth Revision (ICD-9) code; (2) patient residence within Shijiazhuang, including its 8 districts, 11 counties, and 3 county-level cities; (3) patient age >18 years. The final dataset comprehensively includes all AECOPD cases in Shijiazhuang meeting the predefined criteria during the study period. The individual-level hospitalization data used in this study are protected patient information and are not publicly available due to privacy regulations. Researchers interested in accessing the de-identified data may submit a formal application to the Shijiazhuang Medical Insurance Center, with details on the application procedure and expected timeline provided in Supplementary material.

To systematically assess the improvements in air quality and associated health effects following the implementation of pollution control policies, this study compiled daily monitoring data from all air quality monitoring stations in Shijiazhuang. Specifically, 24-h average concentrations were used for PM_2.5_, PM10, SO_2_, CO, and NO_2_, while the daily maximum 8-h moving average concentration (O3-8 h) was applied for ozone. Concurrent daily meteorological data—including mean temperature and relative humidity—were obtained from the Shijiazhuang Meteorological Bureau (http://he.cma.gov.cn/sjz/) to control for potential confounding effects of weather conditions on health outcomes, thereby ensuring data completeness and reliability.

This study was approved by the Scientific Ethics Committee of the Second Hospital of Hebei Medical University (Approval No. 2021-R055).

### 2.2 Statistical analysis

This study employed a comprehensive set of statistical models to quantitatively assess the health benefits associated with improvements in air quality. Descriptive statistics were performed for all variables, and time-series plots were generated to visually illustrate the long-term trends of air quality indicators and AECOPD hospitalization rates from 2017 to 2024, providing a preliminary evaluation of the intervention effectiveness. Additionally, Spearman correlation analysis was conducted to examine the relationships between variables. The strength of correlation was interpreted as follows: |*r*_s_| ≥ 0.6 indicated a strong correlation, |*r*_s_| ≤ 0.3 indicated a weak correlation, and *r*_s_ = 0 indicated no correlation.

The core analysis utilized a Generalized Additive Model (GAM) based on a quasi-Poisson distribution to examine the relationship between daily AECOPD admissions and ambient pollutant concentrations, effectively capturing the complex, non-linear associations between pollution exposure and health outcomes. The structure of the GAM is presented as follows:


$$\begin{array}{l} logE\left[Hospt\right]\;=\;\beta 0\overset{6}{\underset{i=1}{\sum }}\;CB\left(Pollutanti,t;L\;=\;7,\;dflag\;=\;4\right)\\ \;+ns\left(ATt,\;df\;=\;3\right)\;+ns\left(RHt,\;df\;=\;3\right)\\ \;+ns\left(doyt,df=7\times Years\right)+DOW \end{array}$$

where *E*[Hosp_t_] represents the expected number of AECOPD admissions on day *t* (*t* = 1, 2, ..., 2,131); β0 represents the model intercept; Pollutant_i, t_ represents the daily concentration of the *i*-th pollutant (units: μg/m3 for PM_2.5_, PM10, SO_2_, NO_2_, and O3; mg/m3 for CO); CB(·) represents the cross-basis function. We specified a maximum lag period of 7 days (Lag 0–7) *a priori* for the primary analysis. This specific window was chosen based on robust biological plausibility and extensive epidemiological evidence indicating that the acute effects of ambient air pollutants on AECOPD hospitalizations are most pronounced within 1 week following exposure, as consistently reported in previous time-series studies [(5, 12, 56)]. DOW is a day-of-week dummy variable adjusting for weekly healthcare-seeking patterns. To control for potential confounders: long-term and seasonal trends were controlled using the temporal spline ns (*doyt, df* = 7 × Years). The choice of 7° of freedom per year for this spline is a standard approach in long-term time-series analyses to absorb temporal variations and mitigate confounding from slow-varying factors such as demographic shifts or changes in healthcare access (51). Similarly, meteorological variables were adjusted using splines with 3 df per year, consistent with prior studies [(13, 14), Li et al., 2025]. Based on this model, we evaluated the independent effects of the six pollutants on AECOPD admission risk under both single-day and multi-day moving average lag scenarios and plotted the corresponding exposure-response curves. All analyses were conducted using the mgcv and dlnm packages in R software (version 4.5.0). The R code for model construction and sensitivity analyses is publicly available in a GitHub repository (https://github.com/cjs2904100618/AECOPD_Shijiazhuang_AirPollution).

To examine the robustness of our primary findings, we conducted comprehensive sensitivity analyses. First, to assess whether the estimated pollutant-AECOPD associations were unduly dependent on the smoothing of long-term trends, we altered the degrees of freedom (*df* ) for the temporal spline function (testing a range from 3 to 11 df per year, which includes comparisons such as *df* = 4/year and *df* = 10/year against our primary choice of *df* = 7/year) (51). Second, using the pollutant concentrations at their respective optimal lag days identified from single-pollutant models as exposure variables, we simultaneously introduced another pollutant with low correlation (Spearman correlation coefficient |*r*_s_| < 0.6) into the models. By systematically comparing effect estimates between single-pollutant and two-pollutant models, we assessed the stability and reliability of our model results.

To quantify the health benefits derived from air pollution control measures, this study first calculated the annual average concentrations of six major air pollutants to assess their systematic downward trends. Subsequently, by integrating data on daily PM_2.5_ exceedances and applying established health risk assessment formulas, we estimated the number of excess AECOPD cases attributable to PM_2.5_ concentrations exceeding standards and analyzed their interannual variation. Finally, through constructing separate statistical models for each year, we examined the temporal trends in the association strength between major pollutants and AECOPD hospitalizations from 2017 to 2024, thereby systematically evaluating the profound health benefits achieved through pollution control interventions.

## 3 Results

From January 1, 2017, to December 31, 2024, a total of 57,782 AECOPD hospitalizations were recorded in Shijiazhuang. The daily average number of admissions decreased from 27 cases in 2019—a year with higher pollution levels—to 19 cases in 2024. Among these patients, 75% were male, 78% were aged 65 years or older, and hospitalizations were more frequent during the cold season than the warm season. The mean daily ambient air pollutant concentrations were as follows: PM_2.5_ 57.57 μg/m3, PM10 105.90 μg/m3, CO 0.91 mg/m3, NO_2_ 38.81 μg/m3, SO_2_ 13.93 μg/m3, and O3 104.49 μg/m3. All measured pollutant concentrations exceeded the Chinese Grade-I 24-h targets (15 μg/m3 for PM_2.5_ and 30 μg/m3 for PM10), with PM_2.5_ and PM10 falling substantially short of these benchmark values (Table 1).

**Table 1:** Air pollutant concentrations, weather conditions, and daily hospital admissions for acute exacerbation of chronic obstructive pulmonary disease in Shijiazhuang (2017–2024).

**Variable**	**Mean ±SD**	**Min**	**P25**	**P50**	**P75**	**Max**
**Air pollutant concentrations**						
PM2.5 (μg/m^3^)	57.57 ± 47.94	6.00	27.00	42.00	69.00	445.00
PM10 (μg/m^3^)	105.90 ± 72.33	8.00	60.00	89.00	130.00	1,084.00
CO (mg/m^3^)	0.91 ± 0.64	0.20	0.50	0.70	1.00	8.40
NO2 (μg/m^3^)	38.81 ± 20.14	4.00	23.00	35.00	50.00	144.00
SO2 (μg/m^3^)	13.93 ± 14.13	2.00	6.00	10.00	17.00	153.00
O_3_ (μg/m^3^)	104.49 ± 59.02	4.00	58.00	95.00	147.00	310.00
**Meteorological measures**						
AT (°C)	15.20 ± 10.74	−10.30	5.70	16.10	24.90	35.90
RH (%)	55.60 ± 20.15	6.00	40.00	56.00	72.00	100.00
**COPD hospitalizations**						
Total	20 ± 10	1	13	18	24	85
Male	15 ± 7	0	10	14	19	57
Female	5 ± 4	0	2	4	6	33
Age 18–64 years	4 ± 3	0	2	4	6	23
Age ≥65 years	15 ± 8	0	10	14	19	67
Cold season	23 ± 12	1	14	20	29	85
Warm season	17 ± 7	2	12	16	21	45

*Mean* ± *SD* Values are expressed as mean ± standard deviation, Min, minimum; Max, maximum; PM_2.5_, particulate matter with aerodynamic diameter < 2.5 mm; PM10, particulate matter with aerodynamic diameter < 10 mm; CO, carbon monoxide; NO_2_, nitrogen dioxide; SO_2_, sulfur dioxide; O3, ozone; AT, average temperature; RH, relative humidity.

Supplementary Figure 1 displays the time-series patterns of ambient air pollutants and AECOPD hospitalizations from 2017 to 2024. Daily concentrations of all pollutants except O3 showed a declining trend. PM_2.5_, PM10, CO, NO_2_, and SO_2_ concentrations were lowest in summer (June–August) and highest in winter (November–February), whereas O3 exhibited an opposite seasonal pattern. AECOPD admissions also demonstrated an overall decreasing trend. Spearman correlation analysis among air pollutants and meteorological factors revealed positive correlations (*r*_s_ = 0.48–0.90) between PM_2.5_, PM10, CO, NO_2_, and SO_2_, with the strongest correlation observed between PM_2.5_ and PM10 (*r*_s_ = 0.90). In contrast, O3 showed negative correlations with these five pollutants but a strong positive correlation with air temperature (*r*_s_ = 0.85). Detailed results are provided in Supplementary Figure 2.

**Figure 1:**
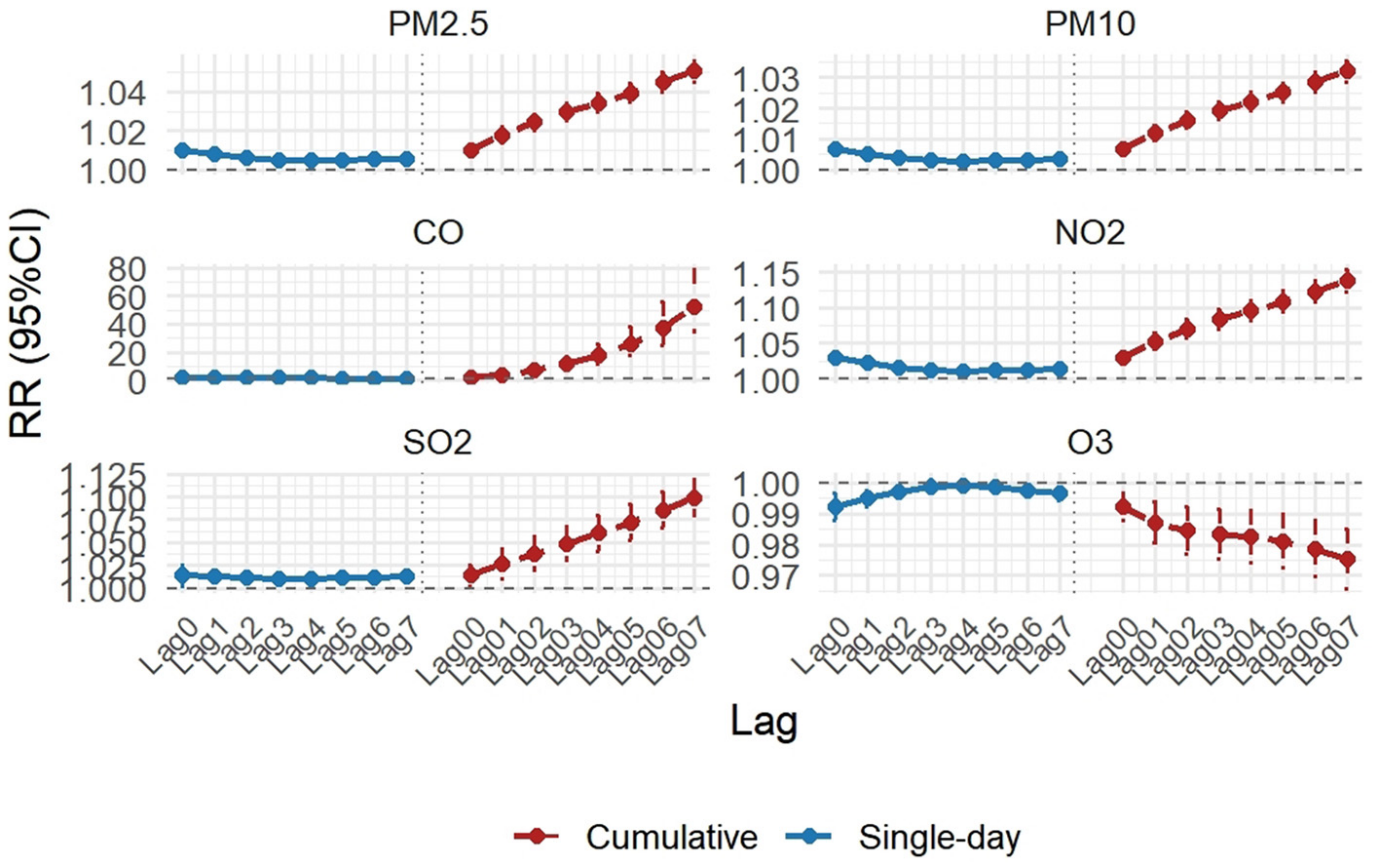
Relative risks (RRs) and 95% confidence intervals (CIs) for AECOPD inpatient admissions associated with a 10 μg/m3 increase in air pollutant concentrations (1 mg/m3 for CO) at different lag days.

Figure 1 and Supplementary Table 1 present the relative risks (RR) and 95% confidence intervals (CI) for daily AECOPD hospitalizations associated with increases in air pollutant concentrations (10 μg/ m3 for all pollutants except CO at 1 mg/m3). After adjusting for day of the week, long-term trends, and meteorological factors, significant positive associations were observed between short-term exposure to air pollutants and AECOPD risk in Shijiazhuang. In single-day lag models, the highest effects for PM_2.5_, PM10, CO, NO_2_, and SO_2_ occurred at lag 0, with RRs of 1.0099 (1.0073–1.0125), 1.0068 (1.0051–1.0084), 1.0798 (1.0567–1.1035), 1.0296 (1.0221–1.0371), and 1.0139 (1.0025–1.0253), respectively. In contrast, O3 showed a negative association at lag 0 (RR = 0.9923, 95% CI: 0.9878–0.9967). In multi-day lag models, significant positive associations persisted for these pollutants, peaking at lag 07 with RRs of 1.0505 (1.0449–1.0560), 1.0320 (1.0286–1.0355), 1.4853 (1.4256–1.5473), 1.1377 (1.1222–1.1534), and 1.0993 (1.0795–1.1195), respectively. Our analysis, focusing on the acute effects within a 7-day exposure window (Lag 0–7) as supported by prior evidence, confirms the substantial threat of air pollution to AECOPD hospitalizations and provides key scientific evidence for evaluating the health benefits of air pollution control policies implemented in Shijiazhuang.

**Figure 2:**
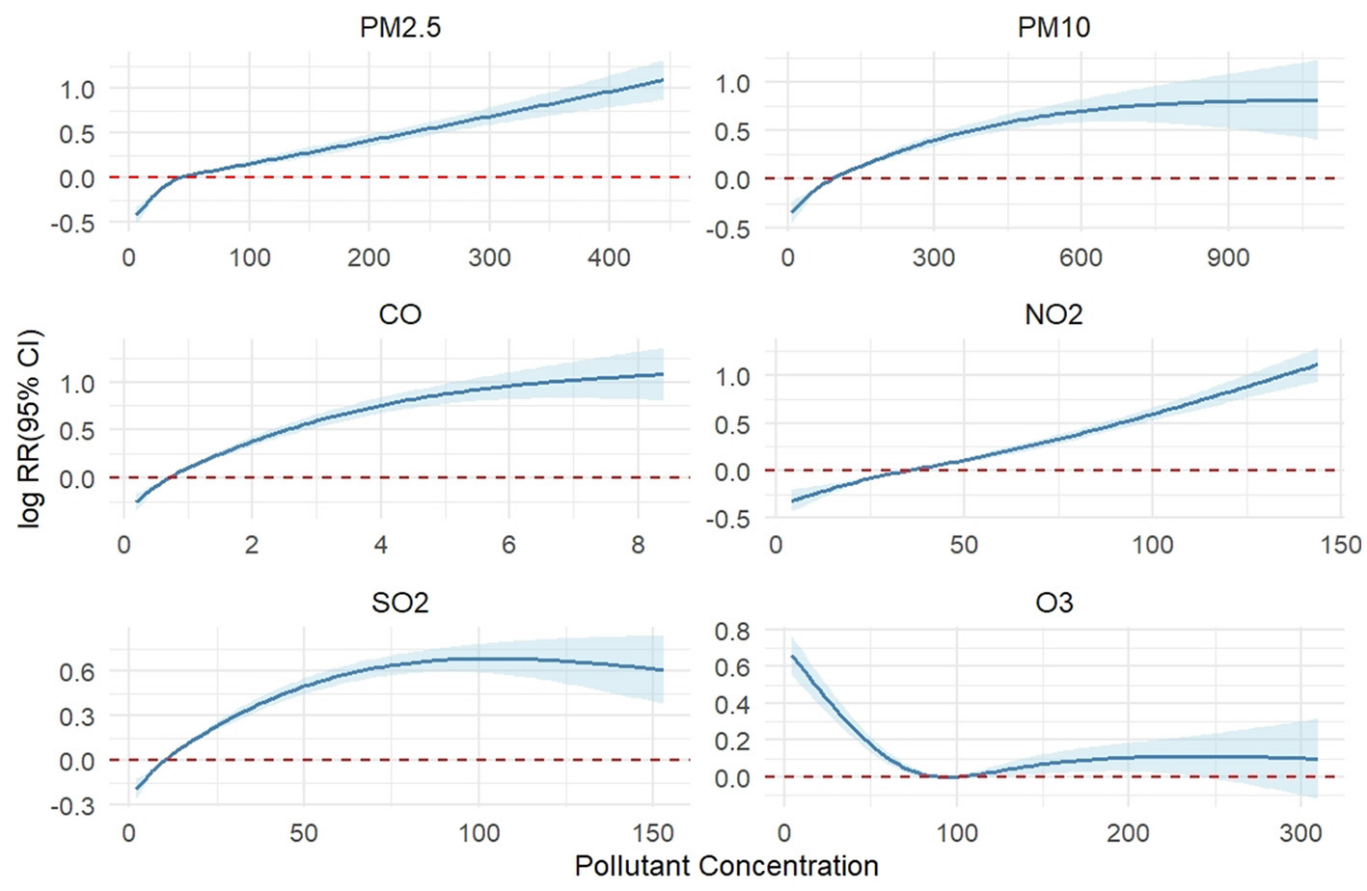
Exposure-response relationships between lag 0 air pollutant concentrations and AECOPD admissions.

Figure 2 presents the exposure-response relationship curves at lag 0. For PM_2.5_, the risk of AECOPD hospitalization increased continuously across the entire observed concentration range (up to 400 μg/m3), with no evidence of a plateau, indicating persistent adverse effects even at high concentrations. In contrast, gaseous pollutants exhibited distinct concentration-response patterns.

In the sensitivity analysis, we first examined whether more flexible spline functions would substantially affect the results by varying the degrees of freedom (*df* = 3–11) for the time trend while maintaining the lag day with the strongest effect for each pollutant. As shown in Supplementary Figure 3, the effect estimates remained generally consistent across different *df* settings, indicating robust model stability. This confirms that the estimated pollutant-AECOPD associations are stable and not unduly dependent on the specific choice of the smoothing parameter for long-term trend control. Second, we assessed collinearity by constructing two-pollutant models. The effects of PM_2.5_, PM10, CO, and NO_2_ remained stable after adjusting for O3. In contrast, the association for SO_2_ changed from a significant positive correlation (RR = 1.0084, 95% CI: 1.0077–1.0091) to non-significant (RR = 0.9975, 95% CI: 0.9853–1.0099) upon adjustment for O3, suggesting that its individual effect is confounded by O3 and should be interpreted with caution (Supplementary Table 2).

**Figure 3:**
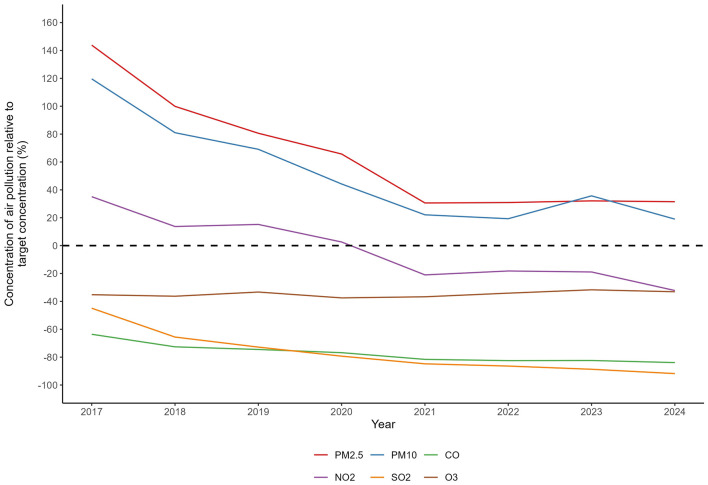
Annual mean concentrations of six criteria air pollutants in Shijiazhuang (2017–2024) as percentages of China's Grade II standards. The dashed line denotes the Chinese Grade II annual concentration target. Values represent the percentage deviation from this target.

The study revealed that from 2017 to 2024, Shijiazhuang experienced a 46% reduction in PM_2.5_ concentrations [from 85.33 μg/m3 (SD 67.16) to 46.04 μg/m3 (SD 33.04)] and a 68% decrease in PM10 concentrations [from 153.75 μg/m3 (SD 90.86) to 83.32 μg/m3 (SD 46.73)]. Improvements in gaseous pollutants were even more pronounced, with SO_2_ decreasing by 85.2% [from 33.07 μg/m3 (SD 25.15) to 4.91 μg/m3 (SD 2.27)], while O3 concentrations showed a slight increase. During the same period, AECOPD hospitalizations exhibited an overall declining trend, with more substantial improvements observed particularly among male and older populations (Supplementary Table 3). Monitoring data indicated systematic declines in the annual average concentrations of all six major air pollutants relative to China's Grade-II Ambient Air Quality Standards, with PM_2.5_ and PM10 showing the most pronounced decreasing trends, and gaseous pollutants including CO, NO_2_, and SO_2_ also demonstrating year-by-year reductions (Figure 3). These marked downward trends are temporally aligned with the implementation phase of the stringent multi-pollutant control policies in Shijiazhuang during the same period. Collectively, these findings indicate sustained improvements in regional air quality over the eight-year period.

Notably, the number of days with PM_2.5_ concentrations exceeding the World Health Organization (WHO) 24-h target (25 μg/m3) in Shijiazhuang decreased from 142 days in 2017 to 56 days in 2024. Concurrently, the number of excess AECOPD cases attributable to daily PM_2.5_ exceedances declined from 8,183 to 5,146 (Table 2), representing a 37.1% reduction and indicating substantial improvement in pollution-related health burden. However, it is noteworthy that even in 2024, when assessed against the more stringent WHO standard, the number of exceedance days remained as high as 265 days, corresponding to 5,146 excess cases. This reveals a persistent gap between China's current air quality standards and the WHO guidelines.

**Table 2:** Number of cases of acute exacerbation of AECOPD advanced by PM2.5 pollution above the expected rates if daily PM2.5 concentrations had not exceeded the standard 24-h targets each year.

**Item**	**2017**	**2018**	**2019**	**2020**	**2021**	**2022**	**2023**	**2024**
**WHO 24-h target (25** μ**g/m**3**)**								
Number of days target not attained	357	333	309	299	264	272	244	265
Number of cases	8,183	8,236	8,559	6,485	4,772	3,224	3,921	5,146
**Chinese grade I 24-h target (35** μ**g/m**3**)**								
Number of days target not attained	316	278	240	231	175	183	185	188
Number of cases	7,360	7,142	7,006	5,028	3,088	2,158	3,175	3,730
**Chinese grade II 24-h target (75** μ**g/m**3**)**								
Number of days target not attained	142	116	87	82	53	59	58	56
Number of cases	3,760	3,609	3,145	2,064	805	640	1,057	1,056

This study conducted a temporal analysis of the associations between six air pollutants and AECOPD hospitalization risk in Shijiazhuang from 2017 to 2024, with all analyses based on the strongest effect estimates (Lag 4) from single-day lag models. The results revealed significant dynamic evolution in the health risks of major pollutants throughout the control period: the effects of PM_2.5_ and PM10 on AECOPD hospitalization risk diminished as control measures intensified. These associations were most pronounced during the initial study period (2017–2018); for instance, in 2017, each 1 μg/m3 increase in PM_2.5_ and PM10 concentrations was associated with 0.36% (RR = 1.0036, 95% CI: 1.0015–1.0056) and 0.28% (RR = 1.0028, 95% CI: 1.0012–1.0044) higher risk of hospitalization, respectively. However, as pollutant concentrations substantially declined, these significant associations gradually disappeared from 2019 onward, with RR values fluctuating around 1 and losing statistical significance. The risk attenuation was most notable for CO, where the hospitalization risk increase per 1 mg/m3 concentration rise declined from 29.3% (RR = 1.2925, 95% CI: 1.0817–1.5443) in 2017 to non-significant levels in later years. NO_2_ exhibited a similarly consistent weakening trend. In contrast, SO_2_ demonstrated no consistent pattern, with its RR values showing substantial fluctuations throughout the study period. Meanwhile, O3 maintained persistent risks without apparent attenuation. These findings indicate that air pollution control in Shijiazhuang has not only significantly reduced pollutant concentrations but has also effectively diminished the unit health risks of major pollutants, demonstrating the dual health benefits of pollution control measures. Detailed results are presented in Table 3.

**Table 3:** Interannual variation in RR values of six air pollutants (2017–2024).

**Pollutant**	**2017**	**2018**	**2019**	**2020**	**2021**	**2022**	**2023**	**2024**
PM2.5 (μg/m^3^)	**1.0036** (1.0015, 1.0056)^*^	**1.0035** (1.0014, 1.0056)^*^	1.0009 (0.9987, 1.0031)	1.0028 (0.9993, 1.0064)	**1.0050** (1.0005, 1.0096)^*^	**0.9890** (0.9826, 0.9954)^*^	0.9963 (0.9921, 1.0006)	0.9993 (0.9949, 1.0037)
PM10 (μg/m^3^)	**1.0028** (1.0012, 1.0044)^*^	**1.0021** (1.0005, 1.0037)^*^	1.0006 (0.9989, 1.0023)	1.0007 (0.9983, 1.0032)	1.0031 (0.9996, 1.0067)	0.9955 (0.9909, 1.0002)	0.9996 (0.9981, 1.0012)	0.9990 (0.9959, 1.0021)
CO (mg/m^3^)	**1.2925** (1.0817, 1.5443)^*^	**1.4173** (1.1763, 1.7077)^*^	1.0478 (0.8648, 1.2696)	1.1110 (0.8082, 1.5273)	**1.5962** (1.0009, 2.5456)^*^	**0.2475** (0.1315, 0.4659)^*^	**0.5309** (0.2898, 0.9725)^*^	0.7990 (0.4492, 1.4212)
NO_2_ (μg/m^3^)	**1.0081** (1.0007, 1.0155)^*^	**1.0107** (1.0042, 1.0172)^*^	1.0012 (0.9940, 1.0085)	1.0010 (0.9928, 1.0092)	0.9982 (0.9874, 1.0092)	0.9866 (0.9726, 1.0009)	0.9895 (0.9765, 1.0027)	0.9946 (0.9813, 1.0081)
SO_2_ (μg/m^3^)	**1.0068** (1.0000, 1.0136)^*^	**1.0113** (1.0018, 1.0210)^*^	0.9947 (0.9786, 1.0111)	1.0229 (0.9962, 1.0504)	0.9825 (0.9506, 1.0155)	0.9939 (0.9419, 1.0489)	1.0159 (0.9587, 1.0766)	1.0303 (0.9662, 1.0987)
O3(μg/m^3^)	**1.0055** (1.0016, 1.0094)^*^	0.9995 (0.9959, 1.0031)	1.0009 (0.9975, 1.0043)	1.0009 (0.9973, 1.0045)	1.0025 (0.9981, 1.0069)	**1.0051** (1.0005, 1.0098)^*^	0.9986 (0.9937, 1.0034)	**1.0048** (1.0000, 1.0096)^*^

Bold values indicate statistically significant RR estimates (*P* < 0.05).

## 4 Discussion

This study quantified the marked attenuation of air pollution and examined the evolving relationships between declining pollutant levels and AECOPD hospitalizations during a stringent multi-pollutant control period in Shijiazhuang (2017–2024), a period marked by the implementation of concrete, large-scale interventions such as coal-to-gas/electricity conversions, industrial boiler retrofits, and high-emission vehicle phase-outs [(15, 16, 50)]. Our principal findings were threefold. First, short-term exposure to multiple air pollutants was significantly associated with increased AECOPD hospitalization risk. Second, Shijiazhuang maintained continued declines in major pollutant concentrations throughout this period. Third, the unit health risks of most pollutants exhibited a weakening trend alongside overall air quality improvement. These results suggest substantial public health co-benefits arising from the implementation of these clean air policies, as evidenced by the concurrent reductions in both pollutant concentrations and their associated health risks, leading to a significant decrease in pollution-attributable disease burden. This provides scientific support for refining future pollution control strategies.

This study confirmed that short-term exposure to pollutants including PM_2.5_, PM10, CO, and NO_2_ significantly increased the risk of AECOPD hospitalization, consistent with findings from numerous domestic and international studies. Research conducted in Shijiazhuang, Beijing, and Chengdu has consistently demonstrated the significant impact of air pollutants on AECOPD admissions (6, 7, 17). Notably, a study in New York reported a distinct lag effect of PM_2.5_, with health impacts emerging gradually over days to weeks following exposure (12), aligning with our finding that the maximum RR values occurred at a cumulative lag of 7 days. Mechanistically, the health hazards of PM_2.5_ primarily stem from its ultrafine particle properties: these particles can penetrate deep into the alveolar region and exert toxic effects through multiple pathways, including systemic inflammation, oxidative stress, dysregulated autophagy and apoptosis, and immune dysfunction [(18, 19, 54, 55)]. Furthermore, the toxicity of PM_2.5_ exhibits complexity, potentially involving synergistic interactions with gaseous pollutants such as SO_2_ and NO_2_. Such multi-pollutant co-exposure may lead to more substantial cumulative damage over multiple days. The acute respiratory risks highlighted in our study are echoed in other regions with high particulate pollution. For instance, in Poldokhtar City, Iran, short-term exposure to elevated PM levels was most strongly associated with adverse respiratory effects among hospitalized patients, underscoring the sensitivity of this population group to air quality fluctuations (20).

Interannual variation analysis revealed cumulative reductions of 46% in PM_2.5_ and 68% in PM10 concentrations in Shijiazhuang over the 8-year study period, a trend consistent with previous research on pollution control effectiveness in this city (15). A national study attributed significant particulate matter reductions in major Chinese cities during 2014–2015 primarily to stringent emission control policies (21). Our study demonstrated a non-linear exposure-response relationship between PM_2.5_/PM10 and AECOPD hospitalization risk (Figure 3). Specifically, no significant association was observed at lower concentration ranges, while the association intensified rapidly with increasing concentrations, exhibiting an approximately linear trend. However, at higher concentrations, PM10 showed evidence of effect saturation, whereas PM_2.5_ demonstrated no clear saturation pattern. This finding aligns with previous research reporting a steep increase in PM10-all-cause mortality association at lower concentrations that plateaued at higher levels (21). The health impacts of PM10 can be severe, extending to mortality in settings of extreme pollution, as evidenced internationally. In Andimeshk City, Iran, exposure to exceptionally high PM10 levels, primarily from dust storms, was significantly associated with increased cardiovascular mortality, illustrating the grave cardiorespiratory burden under such conditions (22). These examples underscore that the detrimental cardiopulmonary effects of particulate matter are a global public health concern, manifesting in region-specific ways. In the southwestern region of Iran, for instance, exposure to dust-derived PM has been linked to a spectrum of adverse outcomes, from asthma and cardiovascular mortality to COPD and lung cancer (23, 24). These results underscore the importance of implementing timely preventive interventions for susceptible populations before ambient PM concentrations peak, to mitigate respiratory exacerbations and avoid hospitalizations. The implementation of clean coal initiatives and stringent industrial emission controls in Shijiazhuang resulted in a remarkable 85% reduction in SO_2_ concentrations. This improvement trajectory closely mirrors findings from a 22-year longitudinal study across four Chinese cities (Lanzhou, Wuhan, Chongqing, and Guangzhou), which documented a dramatic 96.4% decrease in SO_2_ concentrations in Chongqing from 338 μg/m3 in 1995 to 12 μg/m3 in 2017, exceeding even the substantial reduction observed in our study (25). The substantial declines in particulate matter and SO_2_ concentrations are largely attributable to the nationwide implementation of the APPCAP and related remediation efforts. It is important to note that while the observed dramatic reductions are temporally consistent with this policy framework, our ecological study design captures the net health benefit of the integrated control strategy rather than isolating the effect of any single regulatory instrument.

Notably, as concentrations of particulate matter and other gaseous pollutants declined, O3 levels demonstrated an increasing trend—a phenomenon widely reported in other studies (26–28). As a secondary pollutant, O3 formation involves complex mechanisms driven by photochemical reactions involving volatile organic compounds (VOCs) and nitrogen oxides (NO_x_), characterized by a non-linear relationship (29). In Shijiazhuang, VOCs originate mainly from industrial and vehicle emissions (30). In this context, focusing solely on NO_x_ reduction may diminish its inhibitory effect on O3, thereby potentially exacerbating O3 pollution (28). This trend is further supported by observations from multiple regions: for instance, Beijing experienced an annual increase of 1.79 ppbv in O3 from 2004 to 2015 (31), 2 while the Tibetan Plateau recorded an annual rise of 1.71 ppbv during 2015–2019 (32). Nationally, air pollution control measures implemented between 2013 and 2017 reduced NO_x_ emissions by 21%, yet changes in VOC emissions contributed to rising O3 concentrations (33). Additionally, summer high temperatures, intense solar radiation, and regional transport also contribute to elevated O3 levels in Shijiazhuang (34). In contrast to the exposure-response pattern of PM, O3 showed a significant increase in AECOPD hospitalization risk even at low concentrations. As concentrations rose, the risk initially decreased, then slightly rebounded and eventually plateaued (51). The underlying mechanism may involve acute O3 exposure directly inducing airway contraction via neural reflexes (35) and promoting neutrophil infiltration in the airways (36), thereby mediating local and systemic inflammatory responses (37). It should be noted that our current analysis, based on observed ambient ozone concentrations, cannot directly quantify the relative contributions of its major precursors (VOCs and NO_x_) due to the lack of speciated VOC and detailed NO_x_ source-apportionment data for Shijiazhuang. This data gap limits our ability to propose more precise, locally tailored control strategies. In summary, the current single-pollutant control strategy focusing primarily on NO_x_ is insufficient to effectively mitigate O3 pollution. There is an urgent need to integrate VOCs into a coordinated emission reduction framework to address the dual health threats posed by the complex pollution of PM_2.5_ and O3.

Our findings demonstrate that through sustained pollution control, the number of days exceeding PM_2.5_ standards has significantly decreased, accompanied by a corresponding reduction in excess AECOPD cases attributable to short-term pollution exposure. However, it is noteworthy that when assessed against the more stringent WHO guideline (25 μg/m3), Shijiazhuang still faced considerable pollution exposure and associated excess cases even in 2024. This outcome underscores a particular challenge for industrial cities in northern China: despite remarkable achievements in pollution control, achieving year-round compliance with international standards remains difficult due to geographical, meteorological, and industrial constraints. Therefore, for Shijiazhuang and similar cities, we recommend focusing future efforts on promoting clean retrofitting of industrial boilers and expanding the adoption of new energy vehicles to secure further health gains.

Departing from the aggregated analytical approaches common in conventional ecological studies, our innovative construction of annual independent models for the 2017–2024 period has, for the first time, clearly delineated the dynamic trajectory of the association between air pollutants and AECOPD hospitalization risk in Shijiazhuang. The observed attenuation of pollutant-health associations over time aligns with the trajectory of sustained, multi-faceted intervention efforts throughout this period. A fundamental shift occurred in the positive associations of PM_2.5_ and PM10 with AECOPD risk from 2017 to 2024. The significant health risks present during the initial study period completely disappeared by the final years, transitioning to weak, statistically non-significant negative associations. This transformation is likely closely related to Shijiazhuang's continuously advancing particulate matter pollution control policies, including industrial restructuring and energy mix optimization, which have induced structural changes in both particulate concentration and toxic components (52). The risk attenuation was most pronounced for CO and NO_2_, with the substantial decline in CO risk particularly indicating effective control of energy and transportation sources. Both pollutants originate primarily from fossil fuel combustion, especially vehicle emissions and industrial processes (38, 39). The dramatic decline in their health risks likely reflects the positive effects of measures implemented in Shijiazhuang, such as clean energy substitution and industrial denitrification (53). This suggests that the current clean energy transition and technological advances aimed at carbon neutrality are generating substantial and far-reaching co-health benefits. SO_2_ exhibited considerable fluctuation in RR values during the study period. This instability may be associated with SO_2_ concentrations already being at low levels, making their health effects more susceptible to interference from confounding factors such as seasonal meteorological conditions and complex pollution effects (40). Furthermore, with the widespread implementation of coal desulfurization facilities, the atmospheric proportion of SO_2_ has decreased, and its individual health effects may be masked by other secondary pollutants or mixed exposures (41). Unlike other pollutants, O3 maintained relative risks (RRs) fluctuating narrowly around 1.0 throughout the study period, showing no clear risk attenuation trend and even demonstrating borderline statistical significance in 2024 (RR = 1.0048, 95% CI: 1.0000–1.0096). This pattern underscores the unique challenge posed by O3 as a secondary pollutant within the regional complex air pollution context. Its atmospheric concentration is governed by non-linear chemical mechanisms involving precursors (NOx, VOCs), making its control particularly challenging and highlighting the urgent need for enhanced coordinated reduction of VOC emissions (42).

Of particular note, the occurrence of RR values below 1 for certain pollutants in specific years (e.g., PM_2.5_ RR=0.9890 and CO RR=0.2475 in 2022) should not be interpreted as these pollutants conferring a “protective effect” against AECOPD. A more scientifically plausible explanation involves three key considerations. First, the overall improvement in Shijiazhuang's air quality has led to reduced mean pollutant concentrations, substantially weakening the statistical association between exposure and AECOPD hospitalization (43). Second, as concentrations of potent pollutants like CO have dramatically declined, their synergistic pathogenic effects with other pollutants have diminished, statistically manifesting as reduced risk estimates for remaining pollutants in single-pollutant models (44). Finally, enhanced public awareness and adaptive behavioral changes (e.g., reducing outdoor activities during high pollution episodes) may have partially obscured the true exposure-response relationship, resulting in observed risk estimates that are lower than theoretical predictions (45). Additionally, other system-level factors during the study period, especially the COVID-19 pandemic, may have contributed to the observed attenuation and occasional negative associations (46). The implementation of non-pharmaceutical interventions (NPIs), such as mask-wearing, social distancing, and periods of lockdown, likely reduced overall respiratory virus transmission and may have altered healthcare-seeking behaviors for AECOPD (47). Furthermore, potential changes in diagnostic or hospital admission criteria for AECOPD over time cannot be entirely ruled out as contributing factors (48). While our time-series model controlled for broad temporal and seasonal trends, these specific, concurrent system shocks could have further diluted the measured short-term association between air pollution and hospital admissions.

Sensitivity analyses confirmed the robustness of our primary models. In two-pollutant models, the effects of PM_2.5_, PM10, CO, and NO_2_ remained stable after adjusting for O3, whereas the association for SO_2_ disappeared. Given the absence of strong collinearity between SO_2_ and O3 (*r*_s_ = −0.2), this phenomenon may arise from complex mechanisms such as atmospheric chemical interactions or toxicological antagonism. Consequently, multi-pollutant models should be employed when assessing the health risks of SO_2_.

Our study possesses several notable strengths: (1) The analysis is based on an extensive eight-year dataset from Shijiazhuang, a typical heavy industrial city, following the implementation of comprehensive pollution control measures, representing one of the longest and largest time-series analyses in this research domain. (2) We employed generalized additive models (GAM) that effectively captured the non-linear relationships and lagged effects between pollutants and AECOPD hospitalizations (49). The main limitations of our study include: (1) As an ecological time-series study, our analysis relies on aggregate, population-level data. Consequently, we could not adjust for individual-level confounders such as smoking history, occupational exposures, socioeconomic status, or comorbidities. The potential influence of these factors on the observed pollutant-AECOPD associations warrants careful consideration. (2) Although we employed high-degree-of-freedom temporal splines to control for broad temporal patterns, our ecological design cannot separately quantify the contributions of specific factors such as population aging, influenza activity, smoking cessation rates, or particular healthcare policies, nor can it isolate the effect of any single regulatory instrument. Therefore, while the temporal alignment between pollutant reduction and risk attenuation is strongly suggestive, residual confounding cannot be entirely ruled out. (3) Our analysis focused on the acute health effects within a maximum lag period of 7 days, which is well-justified for capturing exacerbation events but may not account for potential longer-term or sub-acute impacts of air pollution exposure. Future studies with different designs could explore health associations over extended time windows.

## 5 Conclusion

This study provides empirical evidence on the health co-benefits accompanying stringent air pollution control in Shijiazhuang during the 2017–2024 period. Our findings indicate that the substantial improvements in air quality observed over this period were associated with a reduced AECOPD hospitalization burden and a marked attenuation in the strength of pollutant-health associations. This dual benefit of reduced pollution levels and attenuated unit health risks highlights the substantial public health gains achievable through sustained, multi-pollutant control. However, the persistent rise in O3 concentrations and its unabated health risk underscore an ongoing challenge, necessitating a shift in strategy. We recommend that future policies prioritize coordinated VOC and NO_x_ emission reduction alongside continued optimization of energy and transportation structures. This integrated approach is essential for achieving sustained improvements in air quality and public health in Shijiazhuang and similar industrial cities.

## Data Availability

The data analyzed in this study is subject to the following licenses/restrictions: The hospitalization data used in this study were provided by the Shijiazhuang Medical Insurance Center and are subject to licensing agreements, therefore they are not publicly available. De-identified data supporting the findings may be made available upon reasonable request and with permission from the data owner (Shijiazhuang Medical Insurance Center) for the purpose of verifying study results. All data processing and usage strictly adhere to the principles of the Declaration of Helsinki and have been approved by the Ethics Committee (2021-R055). Requests to access these datasets should be directed to Xixin Yan, yanxixin@hebmu.edu.cn.
