# Supplementary material for: Evolving relationships between air pollution attenuation and AECOPD hospitalizations during a stringent control period in Shijiazhuang, China, 2017–2024
Source: Front Public Health. 2026 Feb 10;14:1741666. doi: 10.3389/fpubh.2026.1741666 (PMC12929520; doi:10.3389/fpubh.2026.1741666)
Supplement: Supplementary file 1 [file Data_Sheet_1.docx]

Supplementary Material


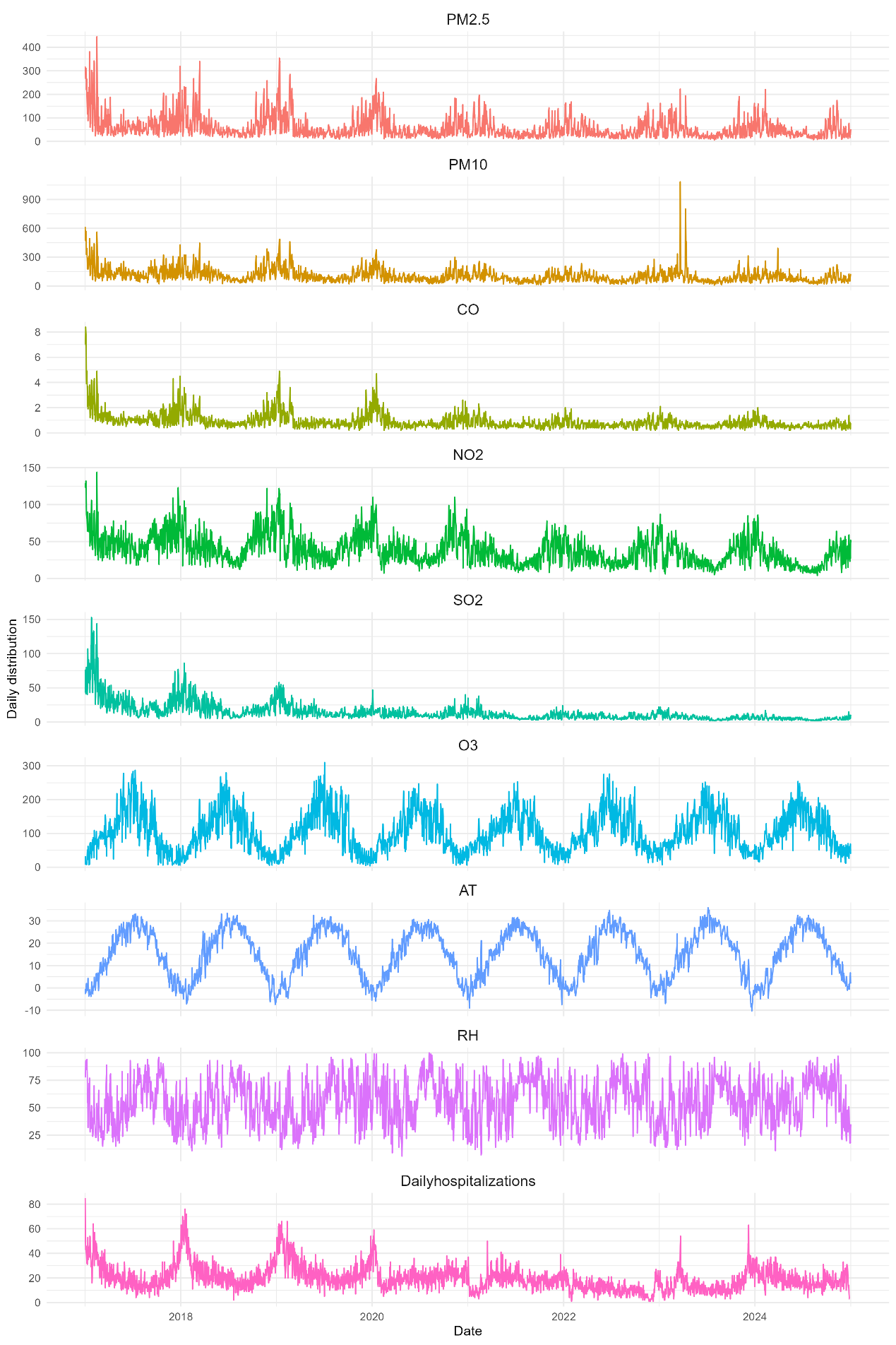


**Supplementary Figure 1.** Distribution of ambient air pollutant concentrations and meteorological variables in Shijiazhuang, China, 2017–2024.


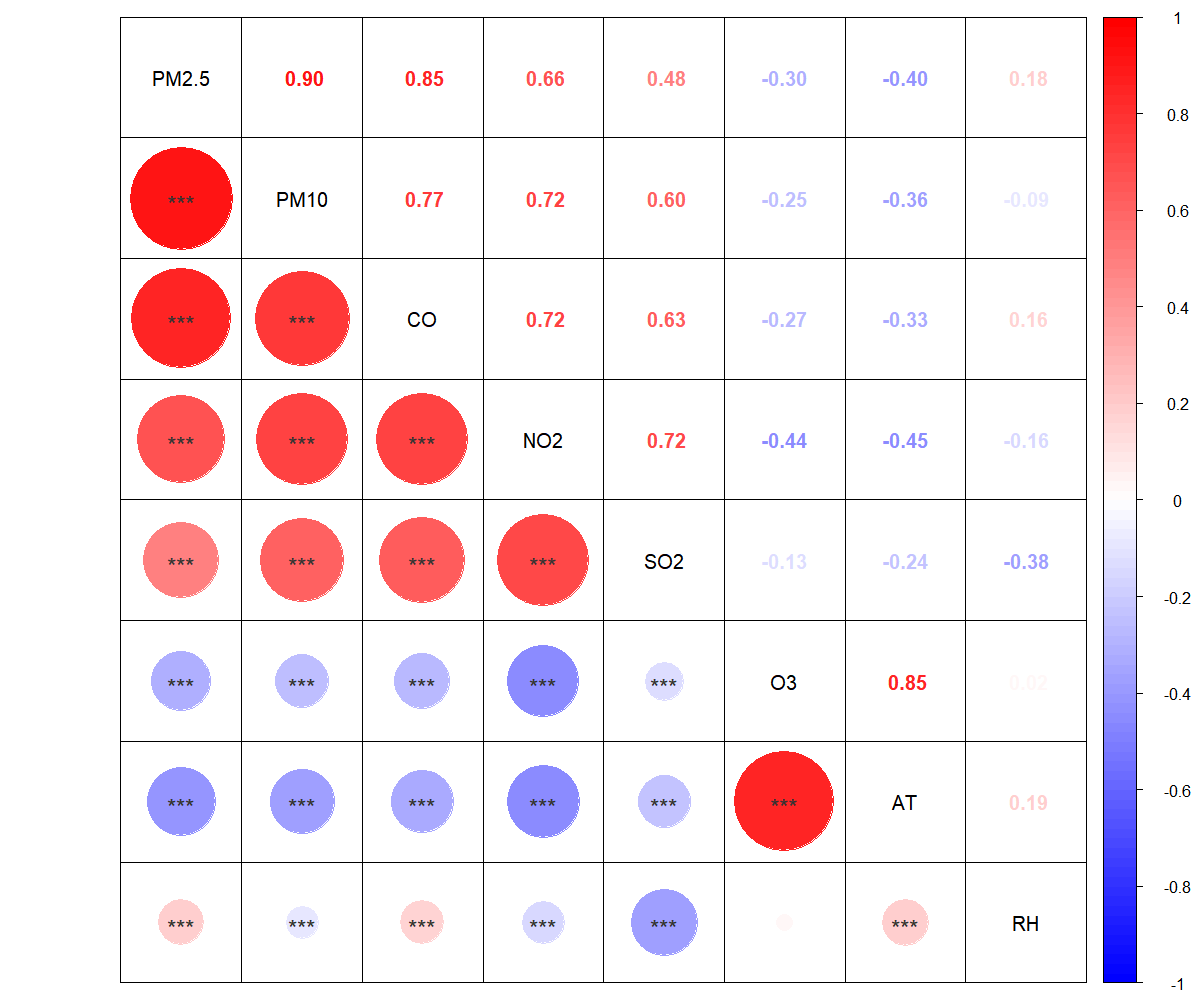


**Supplementary Figure 2.** Correlation matrix of air pollutants and meteorological factors in Shijiazhuang, China, 2015–2024. *AT* Average temperature (℃); *RH* Relative humidity (%)

**Supplementary Table 1.** Relative risks (RRs) and 95% CIs for AECOPD inpatient admissions associated with a 10 μg/m³ increase in air pollutant concentrations (1 mg/m³ for CO) at different lag days

| Lag | PM2.5 | | PM10 | | | CO | | NO2 | | SO2 | | O3 | |
| --- | --- | --- | --- | --- | --- | --- | --- | --- | --- | --- | --- | --- | --- |
|  | RR | 95%CI | | RR | 95%CI | RR | 95%CI | RR | 95%CI | RR | 95%CI | RR | 95%CI |
| Lag0 | **1.0099** | **(1.0073,**  **1.0125)*** | | **1.0068** | **(1.0051,**  **1.0084)*** | **1.0798** | **(1.0567,**  **1.1035)*** | **1.0296** | **(1.0221,**  **1.0371)*** | **1.0139** | **(1.0025,**  **1.0253)*** | **0.9923** | **(0.9878,**  **0.9967)*** |
| Lag1 | **1.0080** | **(1.0066,**  **1.0093)*** | | **1.0053** | **(1.0044,**  **1.0061)*** | **1.0677** | **(1.0555,**  **1.0800)*** | **1.0227** | **(1.0187,**  **1.0267)*** | **1.0124** | **(1.0066,**  **1.0182)*** | **0.9949** | **(0.9924,**  **0.9975)*** |
| Lag2 | **1.0063** | **(1.0052,**  **1.0074)*** | | **1.0040** | **(1.0033,**  **1.0047)*** | **1.0568** | **(1.0469,**  **1.0688)*** | **1.0167** | **(1.0134,**  **1.0200)*** | **1.0112** | **(1.0065,**  **1.0160)*** | **0.9972** | **(0.9953,**  **0.9992)*** |
| Lag3 | **1.0052** | **(1.0038,**  **1.0065)*** | | **1.0031** | **(1.0022,**  **1.0041)*** | **1.0482** | **(1.0355,**  **1.0611)*** | **1.0126** | **(1.0083,**  **1.0168)*** | **1.0106** | **(1.0042,**  **1.0169)*** | 0.9988 | (0.9965,  1.0010) |
| Lag4 | **1.0047** | **(1.0034,**  **1.0060)*** | | **1.0028** | **(1.0020,**  **1.0037)*** | **1.0429** | **(1.0311,**  **1.0547)*** | **1.0110** | **(1.0072,**  **1.0149)*** | **1.0106** | **(1.0049,**  **1.0164)*** | 0.9992 | (0.9971,  1.0012) |
| Lag5 | **1.0049** | **(1.0038,**  **1.0059)*** | | **1.0029** | **(1.0023,**  **1.0036)*** | **1.0399** | **(1.0303,**  **1.0495)*** | **1.0116** | **(1.0084,**  **1.0148)*** | **1.0113** | **(1.0068,**  **1.0159)*** | 0.9986 | (0.9969,  1.0003) |
| Lag6 | **1.0052** | **(1.0039,**  **1.0064)*** | | **1.0032** | **(1.0024,**  **1.0041)*** | **1.0374** | **(1.0263,**  **1.0487)*** | **1.0128** | **(1.0090,**  **1.0166)*** | **1.0123** | **(1.0066,**  **1.0180)*** | **0.9976** | **(0.9956,**  **0.9996)*** |
| Lag7 | **1.0053** | **(1.0040,**  **1.0067)*** | | **1.0034** | **(1.0025,**  **1.0043)*** | **1.0377** | **(1.0217,**  **1.0458)*** | **1.0133** | **(1.0091,**  **1.0175)*** | **1.0129** | **(1.0066,**  **1.0193)*** | **0.9965** | **(0.9943,**  **0.9988)*** |
| Lag01 | **1.0179** | **(1.0140,**  **1.0218)*** | | **1.0121** | **(1.0097,**  **1.0145)*** | **1.1529** | **(1.1164,**  **1.1907)*** | **1.0529** | **(1.0415,**  **1.0644)*** | **1.0265** | **(1.0097,**  **1.0435)*** | **0.9873** | **(0.9805,**  **0.9941)*** |
| Lag02 | **1.0243** | **(1.0200,**  **1.0287)*** | | **1.0162** | **(1.0135,**  **1.0188)*** | **1.2184** | **(1.1762,**  **1.2622)*** | **1.0705** | **(1.0577,**  **1.0834)*** | **1.0380** | **(1.0197,**  **1.0565)*** | **0.9845** | **(0.9769,**  **0.9923)*** |
| Lag03 | **1.0296** | **(1.0252,**  **1.0341)*** | | **1.0193** | **(1.0166,**  **1.0221)*** | **1.2722** | **(1.2318,**  **1.3243)*** | **1.0839** | **(1.0705,**  **1.0975)*** | **1.0489** | **(1.0304,**  **1.0678)*** | **0.9833** | **(0.9753,**  **0.9914)*** |
| Lag04 | **1.0345** | **(1.0298,**  **1.0392)*** | | **1.0222** | **(1.0193,**  **1.0251)*** | **1.3319** | **(1.2824,**  **1.3834)*** | **1.0959** | **(1.0817,**  **1.1103)*** | **1.0601** | **(1.0408,**  **1.0797)*** | **0.9825** | **(0.9741,**  **0.9910)*** |
| Lag05 | **1.0395** | **(1.0346,**  **1.0444)*** | | **1.0252** | **(1.0221,**  **1.0283)*** | **1.3851** | **(1.3321,**  **1.4401)*** | **1.1086** | **(1.0939,**  **1.1235)*** | **1.0721** | **(1.0526,**  **1.0920) *** | **0.9812** | **(0.9724,**  **0.9899)*** |
| Lag06 | **1.0449** | **(1.0398,**  **1.0501)*** | | **1.0285** | **(1.0253,**  **1.0317)*** | **1.4369** | **(1.3813,**  **1.4948)*** | **1.1228** | **(1.1079,**  **1.1379)*** | **1.0853** | **(1.0659,**  **1.1049)*** | **0.9788** | **(0.9698,**  **0.9879)*** |
| Lag07 | **1.0505** | **(1.0449,**  **1.0560)*** | | **1.0320** | **(1.0286,**  **1.0355)*** | **1.4853** | **(1.4256, 1.5473)*** | **1.1377** | **(1.1222,**  **1.1534)*** | **1.0993** | **(1.0795,**  **1.1195)*** | **0.9754** | **(0.9659,**  **0.9850)*** |

“*”P＜0.05


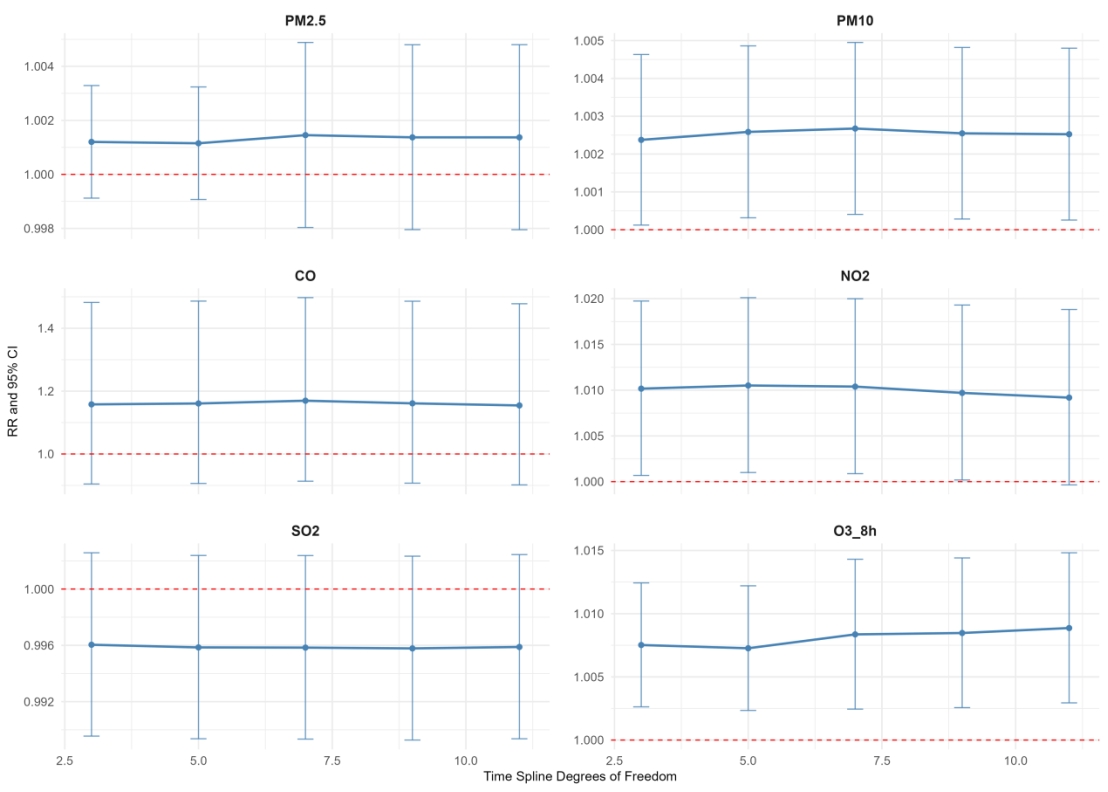


**Supplementary Figure 3. Sensitivity analysis examining the robustness of pollutant-AECOPD associations to varying degrees of freedom (df) for the temporal spline.**
Relative risks (RRs) for key air pollutants across a range of temporal spline df settings (df = 3–11 per year)

**Supplementary Table 2.** Results of two-pollutant model analysis of the effect of air pollutants on AECOPD hospitalizations

| Singel-pollutant | Two-pollutant | RR(%) | 95CI（%） |
| --- | --- | --- | --- |
| PM2.5 |  | 1.0020 | (1.0017,1.0022) |
|  | PM2.5+SO2 | 1.0121 | （1.0080，1.0162） |
|  | PM2.5+ O3 | 1.0033 | （0.9999，1.0068） |
| PM10 |  | 1.0023 | (1.0000,1.0046 ) |
|  | PM10+O3 | 1.0039 | (1.0016,1.0062) |
| CO |  | 1.1884 | (1.1674,1.2097 ) |
|  | CO+O3 | 1.0279 | (1.0022,1.0543) |
| NO2 |  | 1.0084 | (1.0077,1.0091) |
|  | NO2+O3 | 1.0192 | (1.0093,1.0292) |
| SO2 |  | 1.0084 | （1.0077，1.0091） |
|  | SO2+ O3 | 0.9975 | （0.9853，1.0099） |

**Supplementary Table 3.** Trends in Air Pollutant Concentrations, Meteorological Factors, and Hospitalizations for AECOPD during the Study Period (2017-2024)

|  | 2017 | 2018 | 2019 | 2020 | 2021 | 2022 | 2023 | 2024 |
| --- | --- | --- | --- | --- | --- | --- | --- | --- |
|  | Mean±SD | Mean±SD | Mean±SD | Mean±SD | Mean±SD | Mean±SD | Mean±SD | Mean±SD |
| **Air pollutant concentrations** |  |  |  |  |  |  |  |  |
| PM2.5 (μg/m^3^） | 85.33±67.16 | 69.98±52.38 | 63.20±55.57 | 58.00±45.02 | 45.71±34.25 | 45.81±31.00 | 46.23±36.36 | 46.04±33.04 |
| PM10 (μg/m^3^) | 153.75±90.86 | 126.70±73.91 | 118.38±76.08 | 100.96±59.90 | 85.46±46.61 | 83.51±42.32 | 94.97±90.67 | 83.32±46.73 |
| CO (mg/m^3^) | 1.46±1.06 | 1.10±0.61 | 1.02±0.71 | 0.93±0.62 | 0.735±0.335 | 0.70±0.30 | 0.70±0.32 | 0.64±0.27 |
| NO2 (μg/m^3^) | 54.02±20.587 | 45.47±20.56 | 46.07±21.51 | 41.02±19.91 | 31.60±14.77 | 32.71±14.80 | 32.46±16.61 | 27.14±15.24 |
| SO2 (μg/m^3^) | 33.07±25.15 | 20.62±12.73 | 16.33±9.94 | 12.40±5.92 | 9.13±5.39 | 8.16±3.60 | 6.78±3.761 | 4.91±2.27 |
| O3(μg/m^3^) | 103.66±67.22 | 101.86±60.59 | 106.65±68.90 | 99.94±56.68 | 101.35±50.88 | 105.44±57.37 | 109.33±54.76 | 107.11±53.13 |
| **Meteorological measures** |  |  |  |  |  |  |  |  |
| AT (°C) | 15.14±10.69 | 14.76±11.48 | 15.08±10.98 | 14.86±10.32 | 15.39±9.83 | 14.91±10.90 | 15.70±11.14 | 15.66±10.56 |
| RH (%) | 52.95±20.63 | 52.15±19.28 | 53.27±20.71 | 58.34±20.23 | 57.71±21.56 | 56.30±19.79 | 55.07±19.59 | 58.64±18.52 |
| **AECOPD hospitalizations** |  |  |  |  |  |  |  |  |
| Total | 23±12 | 24±12 | 27±11 | 21±8 | 18±7 | 12±6 | 15±8 | 19±6 |
| Male | 16±8 | 17±8 | 19±8 | 16±6 | 14±6 | 9±5 | 12±7 | 15±5 |
| Female | 7±5 | 7±5 | 7±4 | 5±3 | 4±2 | 2±2 | 3±2 | 4±2 |
| Age 18-64 years | 6±3 | 6±3 | 6±3 | 5±2 | 4±2 | 2±2 | 3±2 | 3±2 |
| Age ≥65 years | 17±9 | 18±10 | 20±9 | 17±7 | 14±6 | 9±5 | 12±7 | 15±5 |
| Cold season | 29±12 | 30±14 | 32±13 | 30±14 | 16±7 | 12±7 | 18±10 | 21±7 |
| Warm season | 17±6 | 18±6 | 21±7 | 20±5 | 20±6 | 11±4 | 12±4 | 17±5 |

|  | 2017 | 2018 | 2019 | 2020 | 2021 | 2022 | 2023 | 2024 |
| --- | --- | --- | --- | --- | --- | --- | --- | --- |
| **WHO 24-h target (25 μg/m³)** |  |  |  |  |  |  |  |  |
| Number of days target not attained | 357 | 333 | 309 | 299 | 264 | 272 | 244 | 265 |
| Number of cases | 8183 | 8236 | 8559 | 6485 | 4772 | 3224 | 3921 | 5146 |
| **Chinese grade I 24-h target (35 μg/m³)** |  |  |  |  |  |  |  |  |
| Number of days target not attained | 316 | 278 | 240 | 231 | 175 | 183 | 185 | 188 |
| Number of cases | 7360 | 7142 | 7006 | 5028 | 3088 | 2158 | 3175 | 3730 |
| **Chinese grade II 24-h target (75 μg/m³)** |  |  |  |  |  |  |  |  |
| Number of days target not attained | 142 | 116 | 87 | 82 | 53 | 59 | 58 | 56 |
| Number of cases | 3760 | 3609 | 3145 | 2064 | 805 | 640 | 1057 | 1056 |

**Supplementary Table 4.** Excess AECOPD Cases Attributed to PM2.5 Exceeding Standards

**Supplementary Note: Procedure for Accessing De-identified Hospitalization Data**

The de-identified hospitalization data underlying this study are administrated by the Shijiazhuang Medical Security Bureau and are subject to privacy protection regulations. Researchers may apply for data access through the bureau‘s formal government information disclosure process, as outlined in its official “Guide to Government Information Disclosure”.

**Key Steps for Application:**

**1. Initial Preparation:** Download the official Government Information Disclosure Application Form and guidelines from the bureau's website.

**2. Submission:** Submit the completed application along with required documents (research proposal, institutional approvals, data security plan) to the Bureau Office.

Primary Method: On-site delivery to the address: No. 216, Zhongshan East Road, Shijiazhuang City, 050011, China.

Alternative Method: Mail or fax (Fax: +86-311-86687742).

**Note:** According to the official guide, applications are not accepted via email or telephone. General inquiries can be made by phone at +86-311-86688873.

**3. Review & Decision:** The bureau conducts a formal review. Applicants should anticipate a decision within approximately 1 to 3 months, in line with official response timelines.

**4. Data Provision:** If approved, data will be provided in a secure, de-identified format pursuant to a data use agreement.

**Important Note:** This summary is based on the bureau's publicly available guidelines. Applicants are strongly advised to consult the latest official guide and contact the bureau directly for the most current procedures and requirements.
